# Protective effects of berberine-loaded chitosan/solid lipid nanoparticles in streptozotocin-induced gestational diabetes mellitus rats

**DOI:** 10.3389/ebm.2025.10749

**Published:** 2025-12-12

**Authors:** Yu Liu, Shaik Althaf Hussain, Hua Yue

**Affiliations:** 1 Department of Obstetrics and Gynecology, XD Group Hospital, Xi’an, China; 2 Department of Zoology, College of Science, King Saud University, Riyadh, Saudi Arabia

**Keywords:** berberine, chitosan, gestational diabetes mellitus, rat, solid lipid nanoparticle

## Abstract

Berberine, known as an antioxidant agent, can improve glycemic indices in animal models of diabetes; however, it is clinically limited by poor bioavailability. Nanoparticles show the desirable capacity as delivery platforms for improving the bioavailability of medicinal agents. Here, we aimed to enhance the bioavailability and therapeutic impacts of berberine in streptozotocin (STZ)-induced gestational diabetes mellitus (GDM) rats by its encapsulation into the chitosan-coated solid lipid nanoparticles (SLNs) formulation. Berberine-loaded chitosan/SLN nanoparticles were formulated by the solvent-injection approach followed by a homogenization operation. The particle size, surface charge, and polydispersity index, as well as encapsulation efficiency percent (EE%), *in vitro* stability and berberine release, and *in vivo* pharmacokinetics were studied. Glycemic indices, such as fasting glucose and insulin, oral glucose tolerance, insulin tolerance, and homeostasis model of insulin resistance (HOMA-IR) scores, as well as the activity level of liver antioxidant and pro-oxidant enzymes, were evaluated in STZ-induced GDM rats. The particle size of berberine-loaded chitosan/SLN formulation was detected in the nano-range with high stability and high EE% as well as a sustained-release profile. Berberine nanoparticle treatment could provide a significantly higher oral bioavailability of berberine in experimental rats. Berberine nanoparticles remarkably reversed the altered glycemic indices, body weight, and pro-oxidant/antioxidant balance in STZ-induced GDM rats, with significantly higher effects than free berberine. In conclusion, chitosan-coated SLN nanoparticles firmly enhanced the therapeutic impacts of berberine on STZ-induced GDM, suggesting chitosan-coated SLN nanoparticles as an efficient oral delivery system for enhancing the bioavailability of berberine and, thus, improving its pharmacological impacts.

## Graphical Abstract

**Figure:**
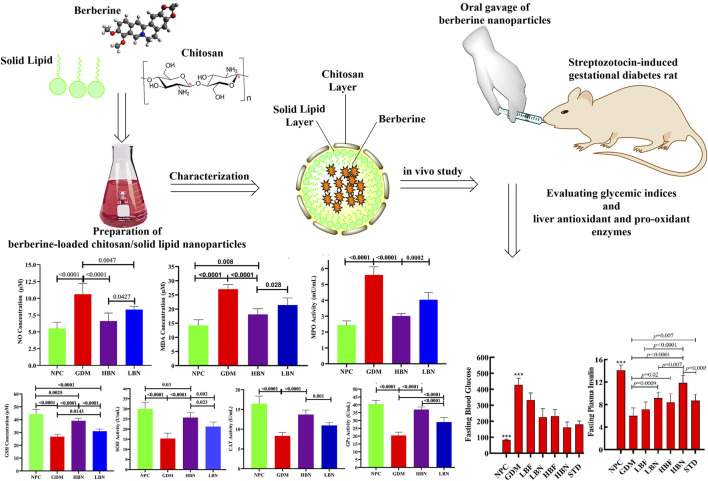

## Impact statement

Chitosan-coated SLN nanoparticles provide an efficient oral delivery system to enhance oral bioavailability of berberine and, thus, improve its pharmacological impacts.

## Introduction

Diabetes Mellitus is a chronic metabolic disease manifested via increased concentration of blood glucose through impaired glucose, protein, and lipid metabolism due to insulin deficiency, which can result from impaired production of insulin by the pancreas gland’s Langerhans beta cells and/or result from insulin resistance by unresponsiveness of the body’s cells to insulin. It is a leading cause of morbidity and mortality, particularly in pregnant women, termed gestational diabetes mellitus (GDM). GDM shows various degrees of glucose intolerance in pregnant women, leading to chronic hyperglycemia that causes long-term injuries, including the development of pre-eclampsia, gestational hypertension, liver damage, and kidney damage. The pathological feature of GDM, like type 2 diabetes mellitus (T2DM), is correlated with both defective insulin secretion and insulin resistance. Notably, normal pregnancy requires elevated glucose production and decreased insulin sensitivity to provide the energy required for the fetus; thus, there is an association between pregnancy and the progression of maternal insulin resistance [1]. During pregnancy, insulin resistance occurs because of the secretion of placental hormones that antagonize insulin. Therefore, during pregnancy, the body needs a high demand for insulin, which increases the load on the pancreatic β-cells, leading to β-cells exhaustion and consequently reduced insulin production [2]. Of note, oxidative stress, an imbalance between the body’s antioxidant defense mechanisms and the generation of free radicals, is an important contributor to the development and pathogenesis of GDM during pregnancy. The increased level of glucose during the GDM condition leads to increased formation of highly toxic oxygen and hydroxyl free radicals through glucose metabolism, such as glucose auto-oxidation, metabolism of methylglyoxal formation, and oxidative phosphorylation [3]. Using antidiabetic drugs (mainly metformin) orally or insulin injection is the main treatment in GDM. However, such treatments are expensive, and long-term use of antidiabetic medications can cause serious adverse impacts on the various organs. In addition, although the insulin therapy can control the blood glucose, it has no therapeutic impact on the function of β-cells and the insulin resistance [4]. On the other hand, targeting oxidative stress may inhibit the GDM-related pathogenesis, suggesting antioxidant agents as potential protective treatment against diabetes complications by reducing free radicals.

Berberine, as a plant-derived medicinal compound, is the major active ingredient detected in the stem, bark, roots, and rhizome of many plants, including barberry (Berberis vulgaris), tree turmeric (Berberis aristata), Oregon grape (Berberis aquifolium), Coptis (Coptis chinensis), and goldenseal (Hydrastis canadensis) [5]. Berberine is mainly known for its antioxidant property, through which it exerts protective impacts on various diseases in preclinical and clinical settings, such as diabetes, cancer, infection, and cardiovascular diseases [6–15]. An increasing body of research has recently shown that berberine can exert effective hypoglycemic activity and, thereby, provide an improving effect on diabetic complications, including diabetic cardiomyopathy, diabetic neuropathy, diabetic nephropathy, diabetic encephalopathy, as well as GDM [11, 16–22]. Interestingly, berberine is found to have a hypoglycemic impact similar to rosiglitazone and metformin in patients with T2DM [23, 24]. Several meta-analyses of clinical trials in T2DM patients revealed that berberine has good safety and a low incidence of total adverse events, and berberine administered alone or in combination with oral hypoglycemic drugs is effective in the treatment of T2DM patients and firmly regulates postprandial blood glucose, fasting blood glucose, and HbA1c [11, 18–20]. Several preclinical investigations have recently shown potential of berberine in the GDM treatment, where it was found to improve glucose tolerance, insulin response, body and fetal weight, placental weight, as well as the number of dead and absorptive fetuses in the experimental models of GDM [21, 25–28]*. B*erberine has been found to exert the glucose-lowering impact by multiple mechanisms, such as elevating glucose uptake, inhibiting gluconeogenesis, enhancing glycolysis, improving insulin secretion and insulin response, and suppressing the action of important enzymes involved in the carbohydrate digestion in the digestive tract (such as α-glucosidase and α-amylase), and improving the anti-oxidant and anti-inflammatory responses [18, 29, 30]. Although berberine has shown a wide range of therapeutic benefits, its oral use is clinically limited because of poor aqueous solubility and intestinal absorption, rapid metabolism by the first-pass effect in the liver and intestine, as well as short biological half-life, which cause low blood concentrations and poor bioavailability. In addition, berberine at high doses causes gastrointestinal side effects, such as cramping and stomach upset, because of its low intestinal absorption and long-term administration [31–33]. Therefore, manufacturing a delivery system to provide a sustained release of berberine is required to improve its bioavailability by reducing the dissolution rate in the gastric environment and increasing the residence time in the intestinal mucus.

Solid lipid nanoparticles (SLNs) have been used as a delivery system to enhance the bioavailability and therapeutic efficiency of natural compounds [34]. In recent years, SLNs have received a significant considerable attention because of their interesting advantages, such as low toxicity and immunogenicity, increasing solubility and bioavailability of both hydrophobic and hydrophilic drugs, triggering pharmacological property of drugs, protecting the drugs against digestive enzymes, having excellent biodegradability and biocompatibility, as well as providing an easy and low-price formulation [35]. The important criteria impacting the *in vivo* efficacy of the oral drug delivery formulations are their integrity and stability in gastrointestinal media. Despite a high stability against digestive enzymes, the low stability of SLNs in the stomach’s acidic pH limits their clinical utilization. For resolving this issue, coating of SLNs by the biopolymers, such as chitosan, with a significant resistance to the acidic media and a high mucoadhesive property, has been employed [36]. Chitosan is a chitin-derived cationic polysaccharide that, under acidic pH, is protonated and strongly binds to the negatively charged SLNs, thereby providing a stable delivery system with the ability to sustainably release an encapsulated drug [37]. Further, when chitosan is coated on the nanoparticle surface, it can improve the absorption of encapsulated drugs via a mucosal surface [38]. Therefore, formulating SLNs with chitosan can protect them against the acidic gastrointestinal medium and enhance their transmucosal delivery, consequently increasing the effective concentration of encapsulated drugs at the absorption site. Of note, chitosan-coated SLN nanoparticles have been found to remarkably improve the oral bioavailability of numerous encapsulated drugs *in vivo* by elevating residence time at mucosa due to their enhanced mucoadhesive property [37, 39–41]. Here, we aimed to bring together biological properties of SLNs and chitosan to manufacture a nanoparticle-based carrier system for oral delivery of berberine, and to determine ameliorating impact of the prepared berberine-loaded nanoparticles on glycemic indices and prooxidant/antioxidant balance in streptozotocin (STZ)-induced GDM rats.

## Materials and methods

### Manufacturing berberine-loaded chitosan/SLN nanoparticles

Berberine-loaded chitosan/SLN nanoformulation was prepared using a previously described solvent-injection method [42] followed by a high-pressure homogenization process [43]. Briefly, berberine (50 mg) and the solid lipid Witepsol 85E (40 mg) were dissolved at 75 °C in 1 mL of a mixed solution (acetone and ethanol in an equal ratio (1:1 v/v)) until a homogenous dispersion appeared. To construct berberine-loaded chitosan-coated SLN nanoparticles, prepared solid lipid/berberine mix was injected into hot double-distilled water (75 °C) containing chitosan (0.5 mg/mL) and a stabilizing surfactant (Pluronic, 2.5 mg/mL). The prepared solution was emulsified by high agitation (25,000 rpm for 4 min at 70 °C) and then homogenized at the same temperature through employing nine homogenization cycles at 750 bars by a high-pressure homogenizer. Immediately after homogenization, the obtained suspension was cooled in an ice-water bath to keep the structure of berberine-loaded chitosan/SLN nanoparticles stable. Finally, the prepared nanoformulation was filtered by a 400 nm filter and then freeze-dried.

### Characterization of the manufactured berberine nano-formulation

#### Physiochemical analysis

Physicochemical indexes of the constructed nanoformulation were studied by measuring the particle size (Z-average diameter), zeta potential (surface charge), and polydispersity index (PDI) by a dynamic light scattering instrument (Zetasizer, UK) at 25 °C.

#### Encapsulation efficiency

To measure the amount of berberine entrapment in the chitosan-coated SLN nano-formulation, the encapsulation efficiency (EE) percentage was evaluated by determining the unloaded (free) berberine concentration through the centrifugal ultrafiltration method, followed by the high-performance liquid chromatography (HPLC) technique. To isolate free berberine, the berberine-loaded SLN/chitosan nano-formulation was centrifuged for 30 min at 23,000 rpm and 4 °C. Afterward, the free berberine amount in the liquid supernatant was determined via HPLC on a Shimadzu system (CBM 20A) equipped with a C18 reverse-phase column (4.6 mm × 25 cm) and a multi-channel UV–VIS detector. To determine the concentration of berberine in the test samples, the HPLC standard curve was made based on the standard solutions containing known concentrations of berberine. Eventually, the nanoparticle EE (%) was determined from the amount of entrapped berberine to the primary loaded berberine. The entrapped concentration of berberine was measured by subtracting berberine amount detected by HPLC from the initially added amount of berberine. The following equation was used to calculate EE (%) [44]: EE (%) = (C_0_ - C)/C_0_ × 100%, where C_0_ is the amount of berberine primary loaded into nano-formulation, and C is the amount of free berberine.

#### *In vitro* stability

The stability of berberine-loaded chitosan/SLN nanoparticles was determined within the simulated gastric fluid (SGF, pH 1.5) and simulated intestinal fluid (SIF, pH 6.8). Nanoparticles were loaded into the SGF and SIF, and then incubated at 37 °C for 2 and 6 h, respectively. Such time intervals were used in accordance with the predicted homing times in the intestine and stomach. Particle size and EE% were evaluated during these time periods.

The SGF medium was prepared by dissolving 2 g/L of sodium chloride NaCl and 3.2 g/L pepsin in deionized water and then adjusting the pH to 1.5 ± 0.2 with 1 M hydrochloric acid (HCl). The SIF medium was prepared by dissolving 6.8 g/L of potassium phosphate monobasic (KH2PO4), 10 g/L of sodium dodecyl sulfate (SDS), and 8400 U/L of 8400 U/L in deionized water and then pH adjusted to 6.8 ± 0.2 with 1 N sodium hydroxide (NaOH) solution [45].

#### *In vitro* drug release

*In vitro* drug release was performed in the SGF and SIF media to simulate the physiological status through oral use. Berberine-loaded chitosan/SLN nanoparticles were incubated at 37 °C through constant shaking (100 rpm) with the SGF or SIF in micro-centrifuge tubes, individually. Nanoparticles were separated through ultrafiltration-centrifugation by Millipore tubes (MWCO = 5 kDa). Eventually, the filtered nanoparticle formulation was analyzed to determine the concentration of berberine by the previously explained HPLC method [46]. The cumulative release calculation method involved determining the percentage of berberine released over time from the nanoformulation, using the following formula [47]:

$$\text{Cumulative percentage release }\left(\%\right)=\frac{\text{Volume}\;\text{of}\;\text{sample}\;\text{withdrawn}\;\left(\text{ml}\right)\;}{\;\text{Bath}\;\text{volume}\;\left(v\right)\;}\times P\;\left(t-1\right)+\text{Pt}$$

Where Pt = percentage release at time “t” and P (t – 1) = percentage release previous to “t.”

#### *In vivo* pharmacokinetic study

Berberine-loaded chitosan/SLN nanoparticles and free berberine were gavaged (50 mg/kg) in two groups (n = 10 rats per group) of female Wistar-Albino rats. Tail vein blood samples were collected and moved into K_3_EDTA tubes at the following times: 0.5, 1, 2, 4, 8, 12, 18, and 24-h after dosing. The plasma was then separated from the blood samples by centrifuging at 3000 rpm for 8 min at 4 °C, and stored in 1.5 mL tubes at −20 °C. Pharmacokinetic parameters, including the area under the berberine plasma versus time curve (AUC), the maximum plasma concentration of berberine after oral administration (C_max_), and the time to maximum plasma concentration of berberine (T_max_), were measured via HPLC analysis of plasma samples [48].

### Gestational rats

Forty-nine female (180 ± 10 g) and seven male (230 ± 10 g) Wistar-Albino rats (8–10 weeks old) were obtained from Takassusi BioMedical, Riyadh, Saudi Arabia. All animal experiments were performed in full accordance with the Animal Welfare instructions and approved by Zhinanzhen Biology Ethics Committee, (No.: A2025000127). All animals were housed in a specific pathogen-free environment in positive pressure rooms at a constant temperature of 22 ± 2 °C and relative humidity of 50–70% with a standard 12/12-h day-night cycle and fed a standard rodent diet and water *ad libitum*. Every possible attempt or action was taken to achieve the lowest suffering. The animals were kept in the laboratory for 1 week to acclimate to the conditions. After acclimatization, vaginal smears were carried out every day to determine the rats’ oestrous cycle, and female rats in the oestrous stage were allowed to mate with male rats by housing them at a 1:1 ratio in individual cages. The next morning, pregnancy in female rats was confirmed by the appearance of sperm in the vagina or by a copulatory plug observed using a microscope, and this day was marked as gestational day (GD) 0 [49].

### Induction of gestational diabetes mellitus

After pregnancy confirmation (GD0), a single dose (50 mg/kg) of streptozotocin (STZ; Sigma–Aldrich) was intraperitoneally injected in the overnight fasted pregnant rats to induce GDM [50]. A group of pregnant rats were intraperitoneally injected with citrate buffer lacking STZ, termed the non-diabetic group. On the fourth day after STZ adminstration, fasting blood glucose (FBG) levels were quantified through the tail incision method with the glucometer (Roche Diagnostic), and pregnant rats suffering blood glucose concentrations more than 180 mg/dL were considered as GDM rats and subjected to further studies. Blood was collected in serum-free tubes through retro-orbital sinus bleeding.

### Experimental design

The experimental study was carried out in seven groups, consisting of seven pregnant rats in each group, allocated by a blinded randomization. Normal Pregnant Control (NPC) group: non-diabetic pregnant rats orally gavaged with SLN/chitosan nanoparticles. Gestational Diabetes Mellitus (GDM) group: GDM rats orally gavaged with SLN/chitosan nanoparticles. LBN group: GDM rats orally gavaged with a Low dose of Berberine Nanoparticles (25 mg/kg/day, LBN). HBN group: GDM rats orally gavaged with a High dose of Berberine Nanoparticles (50 mg/kg/day, HBN). LBF group; GDM rats orally gavaged with a Low dose of Free-Berberine (25 mg/kg/day, LBF). HBF group: GDM rats orally gavaged with a High dose of Free-Berberine (50 mg/kg/day, HBF). Standard (STD) group: GDM rats orally gavaged by metformin (200 mg/kg/day). Berberine-loaded SLN/chitosan nanoformulation was administered to rats by oral gavage once per day from GD4 to GD18. Treatment doses and sample size were used in accordance with already published studies [51–55]. At the final day of the study, the rats were euthanized via intraperitoneal (i.p.) administration of thiopental sodium (30 mg/kg) [56, 57], and the liver tissue was isolated and stored at low temperature (−80 °C) for further experiments.

### Body weight, fasting glucose and insulin, and HOMA-IR

Body weight, as well as the blood glucose level and the serum insulin concentration, were assayed on GD0, GD4, and GD18 after overnight fasting. The body weight of rats was measured by a top loader balance (Thermo Fisher Scientific, Inc.). The FBG level was quantified through the tail incision method with the glucometer (Roche Diagnostics). To measure the fasting insulin levels, the blood samples were obtained from the orbital venous plexus, and plasma samples were isolated by centrifugation at 12,000 rpm for 10 min at 4 °C. The level of fasting insulin was quantified in plasma samples by a rat insulin enzyme-linked immunosorbent assay (ELISA) kit (Termo Scientific). To estimate insulin resistance, the Homeostasis Model of Insulin Resistance (HOMA-IR) was measured by the following formula: HOMA-IR = (Fasting glucose × Fasting insulin)/405. The two main pathophysiological mechanisms of HOMA-IR are insulin resistance and pancreatic cell dysfunction due to insulin resistance [58].

### Oral glucose tolerance test

The oral glucose tolerance test (OGTT) was employed to evaluate glucose tolerance in overnight fasted rats orally given glucose (2 g/kg) 2 weeks after treatment (G18). In brief, glucose solution was orally administered, and the blood level of glucose was detected before and 30, 60, 90, and 120 min after glucose gavage [59]. Data were analyzed as the integrated area under the curve for glucose (AUC_glucose_) and calculated by the trapezoid rule by GraphPad Prism version 9.

### Insulin tolerance test

The insulin tolerance test (ITT) was carried out to evaluate insulin response, reflecting a value of peripheral usage of the blood glucose. After 2 weeks of treatment (G19), insulin (0.8 U/kg, i.p.) was injected to overnight fasted rats. The blood level of glucose was assessed before insulin administration (0 min) and at 30, 60, 90, and 120 min following insulin administration [60]. Results were expressed as AUC_glucose_.

### Hepatic oxidative stress

At the end of the study, the liver tissue was isolated and homogenized at 4 °C in the saline solution (50 mM potassium phosphate, pH 7.0, containing 1 mM EDTA) and centrifuged at 10,000 *g* for 15 min at 4 °C. Afterward, the upper layer of the centrifuged solution was collected for assaying the hepatic status of oxidative stress parameters including super oxide dismutase (SOD; as an antioxidant catalyzing degradation of superoxide radicals into hydrogen peroxide (H_2_O_2_) and O_2_, catalase (CAT; as an antioxidant catalyzing degradation of H_2_O_2_ into H_2_O and O_2_), glutathione (GSH; as an antioxidant scavenging ROS), glutathione peroxidase (GPx; as an antioxidant using GSH as a cofactor to convert hydrogen peroxide and organic hydroperoxides to water and alcohol), malonaldehyde (MDA; as a biomarker of oxidative stress indicating lipid peroxidation), myeloperoxidase (MPO; as an oxidative stress biomarker reflecting liver injury), and nitric oxide (NO; as a biomarker of oxidative stress indicating liver injury), by commercial kits (Cayman Chemical Company, USA) according to the manufacturer’s protocols.

### Statistical analysis

The normality testing (Shapiro-Wilk and Kolmogorov-Smirnov) showed the normal distribution of data and, thus, data were expressed as the mean ± standard deviation (SD). The between-groups significant differences were determined by the unpaired t-test or one-way ANOVA and Tukey-Kramer post-hoc multiple comparison tests (GraphPad Prism software, version 9, San Diego, CA). Data with *p* < 0.05 were statistically considered significant. Data were analyzed by a blinded approach.

## Results

### Physicochemical properties of berberine nanoparticles

The particle size analysis of Berberine-loaded chitosan/SLN nanoparticles showed a considerably narrow particle size and a notably narrow distribution width, verifying a significant dispersion quality. The prepared nanoparticles exhibited average size, surface charge, and PDI of 295 ± 12 nm, +38 ± 1.1 mV, and 0.19 ± 0.09, respectively. Of note, the PDI values < 0.3 confirm a narrow distribution of mean diameter [61]. The surface charge values allow for the prediction of the storage stability of nanoparticles; there are repulsive forces between charged particles, which prevent particle contact and agglomeration. Consequently, charged particles exhibit lower aggregation than neutral particles. The positive surface charge of Berberine-loaded SLN/chitosan nanoparticles is due to the presence of the protonated amino groups in chitosan polymer, which is suitable for obtaining a stable formulation. Additionally, the positive surface charge also confirmed surface coating of the SLN layer by the chitosan polymer. Further, EE% of berberine by chitosan-coated SLN nanoparticles was found to be 88.2 ± 2.4%, and a final lipid-to-drug ratio of 1:0.9.

### *In vitro* stability of berberine nanoparticles

The stability of berberine-loaded chitosan/SLN nanoparticles was evaluated in SGF and SIF media, *in vitro*. As stated in Table 1, the prepared nanoparticles showed a notable stability in SGF and SIF. It can be attributed to the protective effect of multilayer coatings by SLN and chitosan; the SLN component protects nanoparticles against gastrointestinal digestive enzymes, and chitosan can protect the particle stability and integrity against acidic pH. Therefore, SLN and chitosan layers can protect nanoparticles from a harsh gastrointestinal environment, ensuring the robustness and stability of the prepared nanoformulation.

**TABLE 1:** *In vitro* stability of berberine-loaded chitosan/SLN nanoparticles in SGF and SIF media.

Medium	Particle size (nm)			Encapsulation efficiency (%)		
	Pre-incubation	Post-incubation	*p*-value	Pre-incubation	Post-incubation	*p*-value
SGF (pH 1.5)	295 ± 12	267.4 ± 15	0.06	88.2 ± 2.4	63.9 ± 8	0.054
SIF (pH 6.8)	295 ± 12	279.5 ± 19	0.2	88.2 ± 2.4	72.7 ± 6	0.07

### *In vitro* drug release of berberine nanoparticles

**FIGURE 1:**
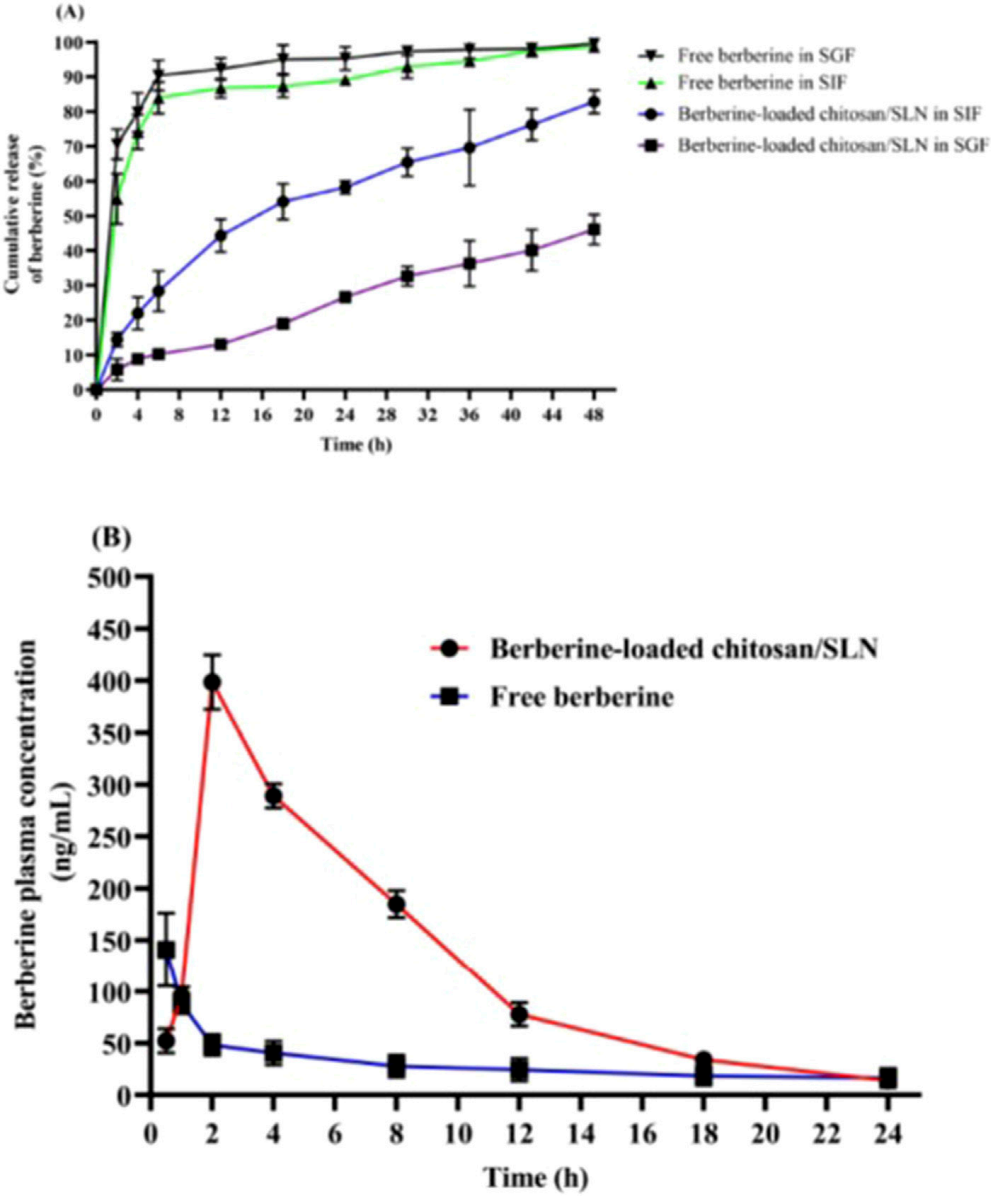
**(A)** *In vitro* release profiles of free berberine and Berberine-loaded chitosan/SLN nanoparticles in simulated gastric fluid (SGF, pH 1.5) and simulated intestinal fluid (SIF, pH 6.8) media. The data are represented as the mean ± SD (n = 3). **(B)** The plasma profile of berberine versus time after oral gavage of free berberine and Berberine-loaded chitosan/SLN nanoparticles in rats. The data are represented as the mean ± SD (n = 10).

The *in vitro* release experiment was carried out in SIF (pH 6.8) and SGF (pH 1.5) media simulating *in vivo* physiological status. Figure 1A shows the profile of berberine release from chitosan-coated SLN nanoparticles in SIF and SGF media. Notably, berberine loaded in chitosan/SLN nanoparticles demonstrated a much slower cumulative release than free-berberine, suggesting the effective protective effect of nanoparticles on berberine against the gastrointestinal harsh acidic environment. The release rates of berberine from chitosan/SLNs nanoparticles were found to be 82.9% ± 3.3 and 46.1% ± 4.3 after 48 h in SIF and SGF media, respectively. The release profile of berberine showed a biphasic pattern during 48 h, including an initiating phase by an early burst release pattern and a late phase by a sustained release pattern. The presence of chitosan is suitable for much slower release rates [62]. Slow releases of Berberine-loaded chitosan/SLN nanoparticles in the SIF and SGF fluids suggest that an adequately high concentration of berberine can be absorbed via the small intestine. Therefore, chitosan/SLN nanoparticles are able to endow a long-acting and effective carrier system for enhancing the intestinal absorption of orally administered berberine.

### *In vivo* pharmacokinetic study

Figure 1B shows increased blood levels of berberine 24 h after oral gavage of berberine-loaded chitosan/SLN nanoparticles. Pharmacokinetic values of Berberine-loaded chitosan/SLN nanoparticles detected in the treated rats are summarized in Table 2. The T_max_ and C_max_ of Berberine-loaded chitosan/SLN nanoparticles were found to be 2 h and 399 ± 26 ng/mL, respectively, whereas significantly lower T_max_ (0.5 h) and C_max_ (141 ± 35 ng/mL) were detected with free berberine. A higher AUC was achieved with Berberine-loaded chitosan/SLN nanoparticles (2,926 ± 75 ng/h/mL) as compared with free berberine (692 ± 74 ng/h/mL). Of note, relative bioavailability exhibited a 2.8-fold increase in the berberine nanoparticle group when compared with the free berberine group. The delayed T_max_ in the berberine nanoparticle group confirmed the sustained berberine release *in vivo*, which is consistent with the *in vitro* release profile results.

**TABLE 2:** Pharmacokinetic parameters of berberine-loaded chitosan/SLN nanoparticles and free berberine in rats after a single oral dose administration.

Parameter	Unit	Berberine nanoparticles	Free berberine	*p*-value
Dose	mg/kg	50	50	
C_max_	ng/mL	399 ± 26	141 ± 35	<0.0001
T_max_	h	2	0.5	
AUC	ng/h/mL	2,926 ± 75	692 ± 74	<0.0001
BA_R_	-	-	2.8-fold	

AUC, area under the berberine plasma concentration-time curve; BA_R_, the relative bioavailability of oral administration of berberine-loaded chitosan/SLN nanoparticles compared with the pure berberine suspension; C_max_, the maximum plasma concentration of berberine after oral administration; T_max_, the time to maximum plasma concentration of berberine.

### Berberine nanoparticles inhibit body weight reduction in the GDM rats

**FIGURE 2:**
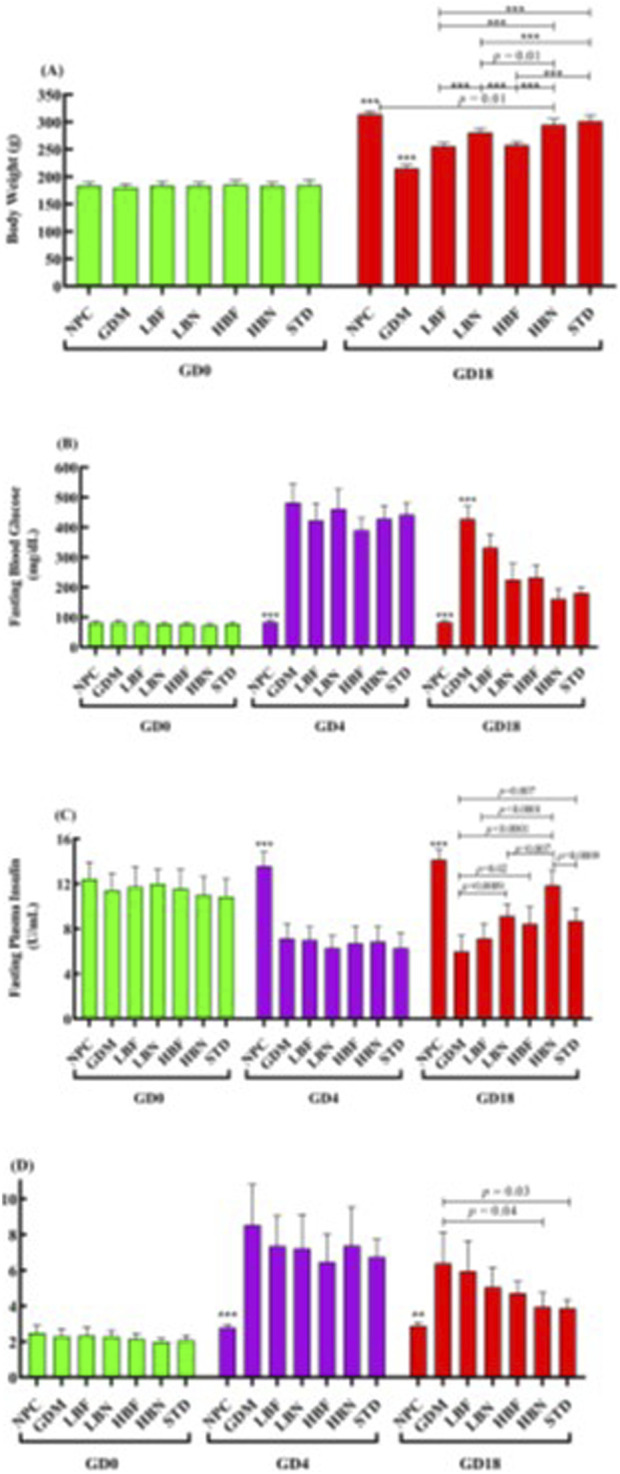
Body weight of rats before pregnancy (GD0) and after 2 weeks treatment (GD18) in the normal pregnant control (NPC) group, gestational diabetes mellitus (GDM) group, GDM rats received a low dose of berberine nanoparticles (the LBN group, 25 mg/kg/day), GDM rats received a high dose of berberine nanoparticles (the HBN group, 50 mg/kg/day), GDM rats received a low dose of free-berberine (the LBF group, 25 mg/kg/day), GDM rats received a high dose of free-berberine (the HBF group, 50 mg/kg/day), and the standard (STD) group including GDM rats received metformin (200 mg/kg/day) **(A)**. Levels of fasting blood glucose **(B)** and fasting plasma insulin **(C)** in NPC, GDM, LBN, HBN, LBF, HBF, and STD groups at GD0, 4 days after STZ injection (GD4), and GD18 **(D)**. The scores of Homeostasis Model of Insulin Resistance (HOMA-IR) in the NPC, GDM, LBN, HBN, LBF, HBF, and STD groups at GD0, GD4, and GD18. Data are represented as the mean ± SD (n = 7). ** and *** indicate *p <* 0.001 and *p <* 0.0001, respectively, for the NPC group or the GDM group when compared with other groups.

The body weight reduction is a major diabetic complication, and is known as an important parameter to estimate diabetes. The body weight of rats before pregnancy (GD0) and after 2 weeks of treatment (GD18) was measured (Figure 2A). The body weight showed no significant difference between all groups of rats before pregnancy at GD0. All group rats showed significant body weight elevation (*p* < 0.0001) at GD18 compared to the initial body weight at GD0. The GDM rats demonstrated a significant body weight reduction (*p* < 0.0001) compared to the NPC group and treatment groups at GD18. GDM rats treated with the low and high doses of berberine nano-formulations (LBN and HBN groups) and of free-berberine (LBF and HBF) demonstrated a significant dose-dependent body weight elevation (*p* < 0.0001) by (65.7 ± 3.6 g and 79.6 ± 4.6 g) and (40.3 ± 3.3 g and 42.6 ± 3.3 g), respectively, compared with the GDM group at GD18. Of note, the body weight showed no significant (*p =* 0.7) difference between the HBN group (295 ± 10.5 g) and the metformin-treated (STD) group (301.6 ± 11 g) at GD18. In addition, there was a significant (*p =* 0.01), but low difference (19 ± 4.6 g) between the body weight of the HBN group (295 ± 10.5 g) and the NPC group (314 ± 6 g) at GD18.

### Berberine nanoparticles improve fasting glucose and insulin in the GDM rats

The changes in the FBG levels are shown in Figure 2B. Four days after STZ injection (GD4), the FBG assessing showed that STZ-injected pregnant rats subjected to a remarkable (*p <* 0.0001) hyperglycemia (482 ± 63 mg/dL) at GD4 as compared with the NPC group (83 ± 5 mg/dL), verifying STZ-induced GDM. The FBG concentration was remarkably elevated in the GDM group at GD18 as compared with the NPC group (*p <* 0.0001). However, the FBG level in the LBN (225 ± 54 mg/dL), HBN (161 ± 35 mg/dL), LBF (332 ± 45 mg/dL), and HBF (232 ± 42 mg/dL) groups was remarkably reduced by 203 ± 26 mg/dL (*p <* 0.0001), 267 ± 20 mg/dL (*p < 0.*0001), 95 ± 23 mg/dL (*p =* 0.007), and 195 ± 22 mg/dL (*p <* 0.0001), respectively, as compared with the GMD group at GD18.

The level of fasting plasma insulin was significantly improved in the GDM rats treated with berberine nano-formulation and free-berberine at GD18 (Figure 2C). The fasting insulin level in the NPC group remained stable at GD0, GD4, and GD18, and it was significantly (*p < 0.*0001) decreased in the GDM group at GD4 and GD18 by 6.5 ± 0.7 U/mL and 8.2 ± 0.7 U/mL, respectively, as compared with the NPC group. Of note, the level of fasting insulin in the LBN (9.2 ± 1 U/mL), HBN (11.8 ± 1.3 U/mL), and HBF (8.5 ± 1.5 U/mL) groups was significantly increased by 3.14 ± 0.67 U/mL (*p =* 0.0009), 5.9 ± 0.7 U/mL (*p <* 0.0001), and 2.5 ± 0.8 U/mL (*p =* 0.02), respectively, at GD18 as compared with the GMD group (6.0 ± 1.4 U/mL). Fasting level of insulin (7.1 ± 1.3 U/mL) was non-significantly increased by 1.14 ± 0.7 U/mL (*p =* 0.47) in the LBF group at GD18 as compared with the GMD group.

As shown in Figure 2D, the HOMA-IR score was significantly elevated by 2-fold (*p =* 0.002) and 1.2-fold (*p =* 0.007) in the GDM group at GD4 and GD18, respectively, as compared with the NPC group, whereas it was reduced significantly in the HBN group by 0.38-fold (*p =* 0.04) and non-significantly in the LBN, LBF, and HBF groups by 0.2-fold (*p =* 0.4), 0.07-fold (*p =* 0.1), and 0.26-fold (*p =* 0.17), respectively, at GD18 as compared with the GMD group. Furthermore, treatment with the high-dose berberine nano-formulation could improve the FBG, fasting plasma insulin, and HOMA-IR score in the HBN group close to those in the STD group treated with metformin.

### Berberine nanoparticles improve glucose tolerance in the GDM rats

**FIGURE 3:**
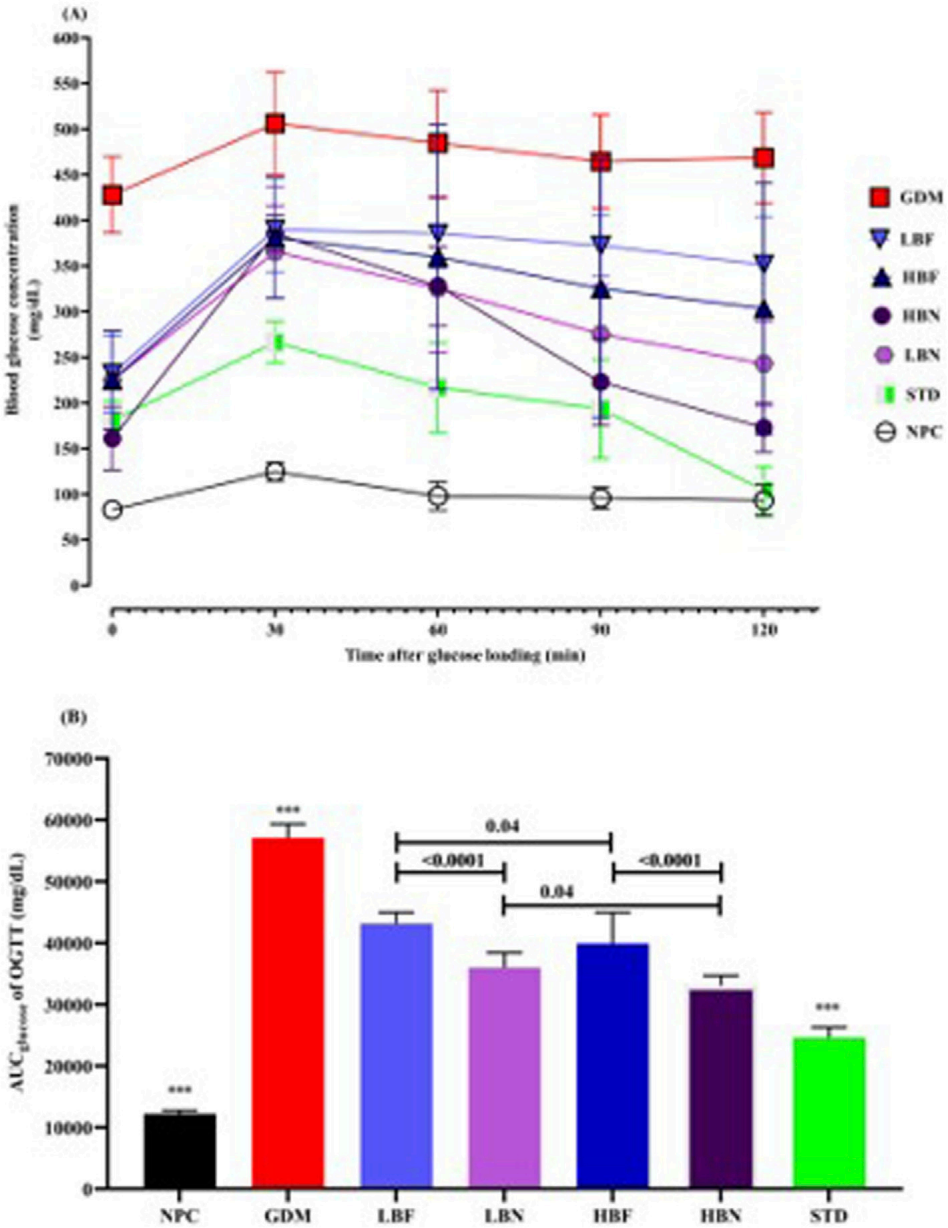
Oral glucose tolerance test **(A)** and corresponding areas under the glucose curve (AUC_glucose_) **(B)** over 120 min after the oral glucose was fed in the NPC, GDM, LBN, HBN, LBF, HBF, and STD groups at GD18. Data are represented as the mean ± SD (n = 7). *** indicates *p <* 0.0001 for NPC group, GDM group, or STD group when compared with treatment groups.

To determine glucose tolerance in the treated GDM rats, an OGTT over 120 min was carried out after 2 weeks of treatment (GD18). 30 min after oral gavage of glucose, a remarkable elevation in the level of blood glucose was detected in the GDM rats compared with NPC rats, indicating a significantly impaired tolerance to exogenously administered glucose. Glucose tolerance ability was remarkably enhanced in the treated GDM rats compared to the untreated GDM rats. A significant reduction in glucose levels was detected after 30 min toward baseline at time 120 in the treated GDM rats (LBN and HBN groups), whereas a consistently high concentration of glucose was found during 30–120 min in the GDM rats. In addition, the blood level of glucose slowly decreased after 30 min in the LBF and HBF groups; however, it did not reach the baseline level at 120 min (Figure 3A).

AUC_glucose_ scores over 120 min of treated GDM rats and GDM rats were remarkably (*p* < 0.0001) higher than NPC rats. Notably, analyzing AUC_glucose_ scores indicated that blood levels of glucose in the different groups of treated GDM rats, including the LBN, HBN, LBF, and HBF groups, were significantly (*p* < 0.0001) reduced up to 37%, 42%, 24%, and 30%, respectively, compared with the GDM group. As the positive control, metformin reduced AUC values up to 57% in the STD group (Figure 3B).

### Berberine nanoparticles improve insulin response in the GDM rats

**FIGURE 4:**
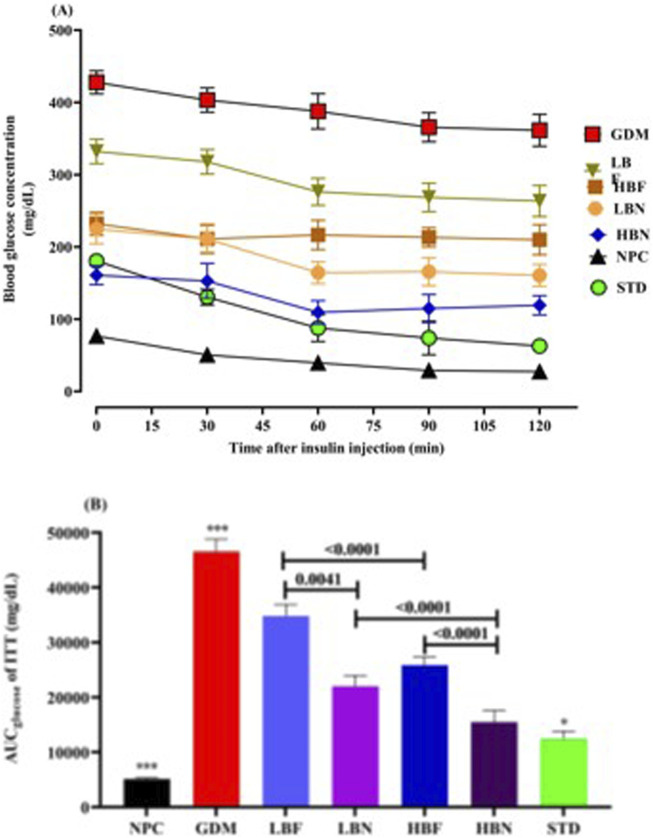
The Insulin tolerance test **(A)** and corresponding areas under the glucose curve (AUC_glucose_) **(B)** over 120 min after the insulin injection in the NPC, GDM, LBN, HBN, LBF, HBF, and STD groups at GD18. Data are represented as the mean ± SD (n = 7). *** indicates *p <* 0.0001 for NPC group or GDM group when compared with treatment groups. * indicates *p* < 0.03 for the STD group when compared with the treatment groups.

To evaluate insulin response in the treated GDM rats, the insulin challenge (0.8 U/kg, i.p.) was conducted at GD19. The blood glucose levels and AUC_glucose_ were significantly (*p* < 0.0001) higher at various time points after the insulin injection in the GDM group compared with the NPC group, showing a remarkably impaired insulin response in the GDM rats. The insulin response ability was significantly enhanced in the treated GDM rats compared to GDM rats. The blood levels of glucose and AUC_glucose_ were lower during ITT in treated GDM rats than in GDM rats (Figure 4). As compared to the GDM group, AUC_glucose_ values in different groups of treated GDM rats, including the LBN, HBN, LBF, and HBF groups, demonstrated significant (*p <* 0.0001) reduction by 52%, 66%, 25%, and 44%, respectively. In addition, AUC_glucose_ values were found to be significantly decreased by 73% (*p <* 0.0001) in the STD group treated with metformin, when compared with the GDM group (Figure 4B).

### Berberine nanoparticles improve the hepatic antioxidant capacity in the GDM rats

**FIGURE 5:**
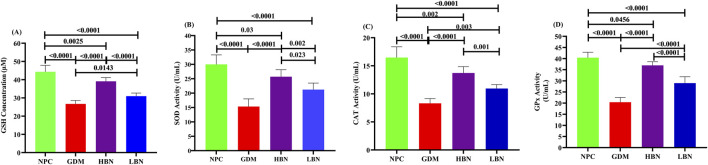
The level of GSH **(A)** and the activity of antioxidant enzymes, including SOD **(B)**, CAT **(C)**, and GPx **(D)** in the NPC, GDM, LBN, and HBN groups at GD18. Data are represented as the mean ± SD (n = 7).

To determine the impact of berberine nanoparticles on the antioxidant status in GDM rats, the concentration of GSH and the activity of antioxidant enzymes GPx, CAT, and SOD were measured in hepatic tissues at the end of the study. As demonstrated in the Figure 5, the level of GSH (26.72 ± 1.86 µM) and the activity of SOD (15.35 ± 3.3 U/mL), CAT (6.93 ± 0.84 U/mL), and GPx (20.43 ± 2.1 U/mL) were significantly decreased by – 38-fold (*p* < 0.0001), – 0.47-fold (*p* < 0.0001), – 0.51-fold (*p* < 0.0001), and – 0.49-fold (*p* < 0.0001), respectively, in the GDM group compared with those (44.34 ± 3.57 µM, 30.01 ± 2.64 U/mL, 16.47 ± 1.92 U/mL, and 40.41 ± 2.49, respectively) in the NPC group. However, when the GDM rats were treated with the high and low doses of berberine nano-formulations (HBN and LBN groups), decreased values of GSH, SOD, CAT, and GPx were significantly and dose-dependently elevated by (0.46-fold (*p* < 0.0001) and 0.16-fold (*p* = 0.01)), (0.67-fold (*p* < 0.0001) and 0.38-fold (*p* = 0.002)), (0.65-fold (*p* < 0.0001) and 0.32-fold (*p* = 0.003)), and (0.81-fold (*p* < 0.0001) and 0.42-fold (*p* < 0.0001)), respectively, in comparison with the GDM group. Additionally, a low but significant difference was found between the mean values of antioxidant parameters in treatment (HBN and LBN) groups and the NPC group.

### Berberine nanoparticles ameliorate the hepatic pro-oxidant load in the GDM rats

**FIGURE 6:**
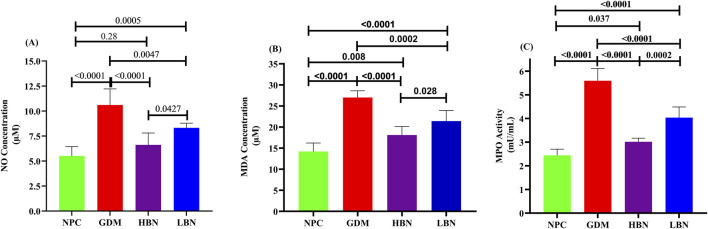
The liver levels of NO **(A)** and MDA **(B)**, as well as the activity of pro-oxidant enzyme MPO **(C)** in the NPC, GDM, LBN, and HBN groups at GD18. Data are represented as the mean ± SD (n = 7).

To further evaluate the effect of the high and low doses of berberine nanoformulations on oxidative stress, the concentrations of NO and MDA, as well as the activity of pro-oxidant enzyme MPO, were measured in the liver tissue at GD19. As shown in Figure 6, the levels of NO (10.61 ± 1.61 µM) and MDA (27.01 ± 1.6 µM) as well as the activity of MPO (5.6 ± 0.51 mU/mL) were significantly increased by 0.92-fold (*p* < 0.0001), 0.9-fold (*p* < 0.0001), 1.3-fold (*p* < 0.0001), and – 0.49-fold (*p* < 0.0001), respectively, in the GDM group compared with those (5.52 ± 0.9 µM, 14.2 ± 2 μM, and 2.44 ± 0.26 mU/mL, respectively) in the NPC group. However, when the GDM rats were treated with the high and low doses of berberine nano-formulations, the increased values of NO, MDA, and MPA were significantly and dose-dependently reduced by (– 0.38-fold (*p* < 0.0001) and – 0.21-fold (*p* = 0.004)), (– 0.33-fold (*p* < 0.0001) and – 0.2-fold (*p* = 0.0002)), and (– 0.46-fold (*p* < 0.0001) and – 0.28-fold (*p* < 0.0001)), in the HBN and LBN groups, respectively, in comparison with the GDM group. Additionally, a significant but low difference (*p* < 0.05) were detected between mean values of pro-oxidant parameters in the treatment (HBN and LBN) groups and the NPC group.

## Discussion

The results from our study indicate that entrapment in chitosan-coated SLN nanoparticles can significantly improve the bioavailability and hypoglycemic effects of berberine in STZ-provoked GDM rats.

Berberine’s ameliorative impacts on the glycemic indices in diabetes mellitus have been well-documented by various preclinical [63–66] and clinical studies [67–71]. There is also growing preclinical evidence indicating the potential of berberine in the GDM treatment. Yang et al. showed that oral gavage of berberine (25, 50, and 100 mg/kg/day from the day before pregnancy till 21st day of pregnancy) could dose-dependently improve body and fetal weight, placental weight, glucose tolerance, and insulin response in the STZ-induced GDM rats [25]. Consistently, Li et al. showed that orally administrated berberine (100 mg/kg/day, gavaged from the 7th to 20th day of pregnancy) could significantly improve maternal insulin response and body weight, placental and fetal weight, as well as the number of dead and absorptive fetuses in the high fat diet-induced GDM rats compared with the GDM rats without berberine treatment [26]. Similarly, other preclinical studies also reported the anti-diabetic impacts of berberine in GDM [21, 27, 28]. However, the poor hydrophilicity and bioavailability of berberine limit therapeutic efficiency after oral administration. Of note, berberine has to be repeatedly used at high doses (0.9–1.5 g/day) once prescribed to patients with diabetes [23]. Though berberine at high doses effectively ameliorates hyperglycemia, it leads to considerable gastrointestinal adverse impacts that limit its clinical use. Thus, improving the oral bioavailability of berberine not only elevates its anti-hyperglycemic impact but also decreases gastrointestinal adverse impacts. To improve berberine bioavailability, numerous investigations have developed berberine nanoformulations and showed their improved effectiveness for treating various diseases, such as diabetes [72, 73]. Based on our current knowledge, there is no published report showing the potential of berberine nanoformulations in GDM treatment. Here, we first studied the anti-hyperglycemic effects of berberine-loaded chitosan/SLN nanoparticles in the STZ-induced GDM rats. STZ induces a diabetes model identified by pancreatic β-cells’ damage, causing insulin deficiency and consequent hyperglycemia and body weight reduction [74]. We found a significantly increased blood glucose concentration and body weight loss in STZ-administered pregnant rats (GDM group), when compared with normal pregnant rats (NPC group). Two weeks’ daily oral gavage of free-berberine (50 and 100 mg/kg/day) could dose-dependently reverse the STZ-induced insulin deficiency, hyperglycemia, and body weight loss in the GDM rats, which were consistent with the above-mentioned studies [21, 25–28]. Of note, we indicated that the ameliorating effects of berberine on glycemic indices and body weight loss were significantly improved when encapsulated in chitosan-coated SLN nanoparticles. The low (25 mg/kg/day) and high (50 mg/kg/day) doses of berberine nanoparticles were found to significantly increase the reduction of FBG levels by 2.1-fold and 1.4-fold in the GDM rats, respectively, when compared with the equal doses of free-berberine. As shown by OGTT analysis, reduction of the blood glucose levels at the given times after glucose challenge in free-berberine- and berberine nanoparticle-treated GDM rats showed a 0.54-fold and 0.4-fold higher glucose tolerance in the GDM rats treated by the low and high doses of berberine nanoparticles, respectively, than those treated by free-berberine. The present findings are similar to other studies showing the enhanced efficacy of other berberine nanoformulations in reducing the elevated levels of FBG in STZ-induced diabetic rodents [75, 76]. Such a reduction of blood glucose levels might be attributed to elevated peripheral utilization of glucose. Our results from evaluating fasting insulin suggest that this can be due to the inducing effect of berberine on insulin secretion by pancreatic cells. Notably, the level of fasting plasma insulin was remarkably reduced in the untreated STZ-induced GDM rats compared with NPC rats, showing impaired insulin secretion. Interestingly, berberine nanoparticle-treated GDM rats demonstrated a significant elevation in fasting insulin levels, where a 2.75-fold and 2.4-fold elevation in fasting insulin was found in GDM rats treated by berberine nanoparticles at the low and high doses, respectively, in comparison with those treated by free berberine. However, low-dose free berberine showed no significant elevation in fasting insulin levels in GDM rats, whereas the high-dose could exert a significant improving effect. Mechanistically, berberine can enhance peripheral usage of glucose, at least in part, through promoting insulin production via pancreatic cells. Zhou et al. reported that berberine can significantly elevate islet area, the number of β-cells, the insulin secretion, and the ratio of pancreas to body weight in diabetic rats [77]. Leng et al. found that berberine dose-dependently induces insulin secretion in HIT-T15 cells and mouse islets [78]. Consistently, Wang et al. showed that berberine causes an insulinotropic effect in rat islets [79]. It has also been found that berberine can inhibit β-cell apoptosis and induce insulin release [80, 81]. Mechanistically, berberine has been shown to induce insulin secretion by elevating the levels of the incretin hormone glucagon-like peptide-1 (GLP-1) that participates in the survival of pancreatic cells. GLP-1, by activating adenylate cyclase that converts ATP into cyclic adenosine monophosphate (cAMP), triggers the epac protein and PKA signaling, leading to an elevation in the intracellular level of Ca^2+^. This promotes the translocation and secretion of insulin granules [82–85].

Besides increasing insulin secretion, results from the ITT’s insulin challenge and *the* HOMA-IR scores suggest that berberine might increase peripheral glucose uptake in STZ-induced GDM rats through enhancing the sensitivity/response of the body’s cells to the insulin effect. Notably, ITT assessment revealed that, after insulin administration, untreated GDM rats were unable to significantly reduce the blood glucose, indicating that GDM rats suffered from a peripheral response deficiency to insulin and thus were unable to utilize exogenously injected insulin to drop the increased levels of blood glucose. However, free berberine and berberine nanoparticles remarkably decreased blood glucose in GDM rats after insulin injection, while 1.1-fold and 0.5-fold reductions were detected in GDM rats that received berberine nanoparticles at low and high doses, respectively, in comparison with those treated by free berberine. Supporting this, the HOMA-IR scores revealed that insulin resistance was significantly reduced by 2.85-fold and 1.5-fold in GDM rats treated with the low and high doses of berberine nanoparticles, respectively, when compared with free-berberine, further verifying an improved response of the body’s cells to the insulin effect in the berberine nanoparticle-treated GDM rats.

During the insulin resistance condition, there is a dysregulation in the insulin response signaling pathway, the Protein Kinase B (Akt)/Phosphoinositide 3-kinase (PI3K)/Insulin receptor substrate 1 (IRS-1). In improving insulin resistance, mechanistically, berberine can induce insulin response by activating Akt through promoting the 5′-adenosine monophosphate-activated protein kinase (AMPK) signaling as an energy-sensing pathway that is activated/inactivated in accordance with the cellular AMP/ATP ratio [82, 86, 87]. Berberine induces AMPK by promoting phosphorylation of Thr172 on the α subunit of AMPK [88–90], as well as by elevating the ratio of cellular AMP/ATP through suppressing the generation of the mitochondrial ATP [91, 92]. Berberine by AMPK can activate Akt that induces the expression and translocation of glucose transporter type-4 (GLUT4) to the cellular membrane, thus, glucose can be uptaken and utilized by the cell, causing increased glucose uptake and usage by insulin-resistant cells [82].

Furthermore, weight reduction is the main property of human T2DM and STZ-induced diabetes because of degradation of structural proteins and muscle injury, as well as worsened lipolysis and increased lipid peroxidation, which are complications of insulin deficiency [93]. Here, we found that berberine nanoformulation and free berberine treatment could significantly inhibit the body weight reduction in the STZ-induced GDM rats, where a 1.6-fold and 1.9-fold elevation of the body weight was found in the GDM rats treated by low and high doses of berberine nanoparticles, respectively, when compared with those treated by the equal doses of free berberine. Similarly, Yang et al. reported the preventive impact of berberine against body weight loss in the STZ-induced GDM rats [25].

Increased oxidative stress, resulting from an imbalance of free radicals producing and scavenging, is another common characteristic in the diabetes onset and development [94–96]. The results of the present investigation showed that berberine nanoparticles can significantly enhance hepatic antioxidant capacity in the STZ-induced GDM rats through increasing the concentration of GSH and the activity of antioxidant enzymes GPx, CAT, and SOD. Further, berberine nanoparticles could significantly reverse the increased levels of NO and MDA, as well as the enhanced activity of pro-oxidant enzyme MPO in STZ-induced GDM rats. Of note, the difference between the berberine nanoparticle-treated GDM rats (HBN/LBN groups) and the NPC rats was found to be small but sufficient. Mechanistically, berberine has been found to upregulate uncoupling protein 2 (UCP2) as a mitochondrial inner membrane protein that has a significantly negative association with ROS generation and oxidative stress [82].

The better protection against diabetes in STZ-induced GDM rats by berberine-loaded chitosan/SLN nanoparticle treatment than free berberine treatment can be attributed to a higher oral bioavailability of berberine achieved by chitosan-coated SLN formulation. After entrapment in chitosan/SLN nanoparticles, berberine showed an elevated level of the plasma peak, a postponed peak time, and an increased AUC in rats, confirming an improved oral bioavailability of berberine. The improved bioavailability of berberine can be attributed to the physicochemical features of berberine-loaded chitosan/SLN nanoparticles, as discussed in the following. The *in vitro* stability and drug release assessment demonstrated that berberine nanoparticles are stable in the SGF condition and show a sustained release profile in the SIF condition. Further, the chitosan moiety provides a hydrophilic surface layer that enables nanoparticles to form strong hydrogen bonds to the intestinal mucosal surface and, thus, enhances the mucoadhesion. The supporting investigations reported the enhanced intestinal uptake of compounds entrapped in chitosan-coated SLN nanoparticles, which was found to be attributed to hydrophilic and ionic interactions [37, 39]. These nanoparticles also showed a positive surface charge that causes an easy attachment to the intestinal cell membrane with the negative charge, leading to an enhanced uptake by intestinal cells [97]. Moreover, particle size can directly influence the cellular uptake of nanoparticles [98]. Berberine-loaded chitosan/SLN nanoparticles demonstrated a nanosize that facilitates an efficient cellular uptake. A phenomenon known as membrane wrapping, which demonstrates how a membrane may enclose a molecule, can explain the size-dependent uptake of nanoparticles. The nano size of berberine-loaded chitosan/SLN particles is also suitable for invisibility in the reticuloendothelial system and long-term circulation in the bloodstream [98, 99].

## Conclusion

We synthesized chitosan-coated SLN nanoparticles to deliver berberine and evaluate their antidiabetic impacts in STZ-provoked GDM rats. The stable and nano-sized particle, high EE%, good *in vivo* stability, and sustained berberine release features are shown by berberine-loaded chitosan/SLN nanoparticles. Compared with free berberine treatment, berberine nanoparticle treatment could provide a significantly higher oral bioavailability of berberine in experimental rats. Chitosan-coated SLN nanoparticles can significantly enhance the protective effect of berberine against hyperglycemia in the STZ-induced GDM rats. Notably, the improved bioavailability of berberine-loaded chitosan/SLN nanoparticles is a possible reason supporting the improved therapeutic impact of berberine in the STZ-induced GDM rats. In conclusion, chitosan-coated SLN nanoparticles provide a suitable delivery system to enhance the oral bioavailability of berberine and, thus, improve its pharmacological impacts.

## Data Availability

The data used to support the findings of this study are available from the corresponding author upon request.
