# Correction: Protective effects of berberine-loaded chitosan/solid lipid nanoparticles in streptozotocin-induced gestational diabetes mellitus rats

**DOI:** 10.3389/ebm.2026.10958

**Published:** 2026-08-03

**Authors:** EBM Production Office

**Affiliations:** Frontiers Media SA, Lausanne, Switzerland

**Keywords:** berberine, chitosan, gestational diabetes mellitus, rat, solid lipid nanoparticle

In the main article, the name of the ethics committee was erroneously given as “the Institutional Ethics Committee and Research Advisory Committee of the King Saud University, Saudi Arabia” and the location that the rats were obtained from was erroneously given as “the laboratory animal research center of the College of Science, King Saud University, Saudi Arabia”.

A correction has been made to the **Material and Methods section,** subsection ‘Gestational rats’. The correct paragraph appears below:

“Forty-nine female (180 ± 10 g) and seven male (230 ± 10 g) Wistar-Albino rats (8–10 weeks old) were obtained from Takassusi BioMedical, Riyadh, Saudi Arabia. All animal experiments were performed in full accordance with the Animal Welfare instructions and approved by Zhinanzhen Biology Ethics Committee, [No.: A2025000127.]”

The original version of this article has been updated.

